# Rectus femoris muscle mass evaluation by ultrasound: facilitating sarcopenia diagnosis in pre-dialysis chronic kidney disease stages

**DOI:** 10.6061/clinics/2018/e392

**Published:** 2018-10-25

**Authors:** Viviane Angelina de Souza, Dílmerson Oliveira, Eduardo Neumann Cupolilo, Carolina Souza Miranda, Fernando Antônio Basile Colugnati, Henrique Novais Mansur, Natália Maria da Silva Fernandes, Marcus Gomes Bastos

**Affiliations:** I Faculdade de Medicina, Universidade Federal de Juiz de Fora, Juiz de Fora, MG, BR; II Campus Rio Pomba, Instituto Federal de Educacao Ciencia e Tecnologia do Sudeste de Minas, Rio Pomba, MG, BR

**Keywords:** Sarcopenia, Ultrasound, Tomography, Renal Insufficiency, Chronic

## Abstract

**OBJECTIVES:**

We evaluated the validity and reliability of ultrasonography measurement of rectus femoris cross-sectional area compared to computed tomography in patients in pre-dialysis chronic kidney disease and analyzed the association between these measurements and the diagnosis of sarcopenia.

**METHODS:**

One hundred patients with nondialysis chronic kidney disease were evaluated. Sarcopenia was defined using the criteria of the Foundation for the National Institutes of Health Sarcopenia Project (FNIH). The rectus femoris cross-sectional area was evaluated using ultrasonography and computed tomography.

**RESULTS:**

The prevalence of sarcopenia was 29% according to the FNIH criteria. The difference in mean rectus femoris cross-sectional area by ultrasonography and computed tomography was 3.97 mm, with a strong correlation between the two methods (*p*<0.001). Bland-Altman plot analysis showed good agreement between computed tomography and ultrasonography. Rectus femoris cross-sectional area was significantly correlated with muscle strength (r=0.300, *p*=0.002), lean body mass in the upper limbs (r=0.286, *p*=0.004), and lean body mass in the lower limbs (r=0.271, *p*=0.006). The prevalence of sarcopenia was 12% (n=12) based on the definition of low muscle mass according to ultrasonography of the rectus femoris cross-sectional area.

**CONCLUSION:**

Ultrasonography was demonstrated to be a valid and reliable method for evaluating the rectus femoris cross-sectional area in patients in pre-dialysis chronic kidney disease.

## INTRODUCTION

Sarcopenia is defined as an age-related progressive loss of muscle mass, coupled with a decline in muscle strength and physical performance 1,2. It is associated with impaired health status, mobility disorders, increased risk of falls and fractures, difficulty in performing activities of daily living, disability, loss of independence, and increased risk of death 1-3.

Among individuals with chronic kidney disease (CKD), sarcopenia may affect approximately 37% of dialysis patients 1,2,4. The prevalence in patients in pre-dialysis CKD stages ranges from 5 to 9% 5. Muscle mass loss in this population is associated with greater morbidity and mortality as well as an increase in cardiovascular complications and protein-energy wasting (PEW) syndrome 1,2,6,7. One potential important cause of sarcopenia in this population is a low-protein diet for the conservative management of CKD in pre-dialysis stages 8. Thus, early identification of sarcopenia and evaluation of possibly modifiable risk factors are of paramount importance in this patient population 1.

In recent years, varying definitions of sarcopenia have been proposed, but no consensus exists regarding its characterization 1,2. The European Working Group on Sarcopenia in Older People (EWGSOP) proposed a set of criteria in 2010 that is widely used in clinical practice and research 1-3. Recently, new definitions have been proposed by the Foundation for the National Institutes of Health Sarcopenia Project (FNIH) 9. The criterion proposed by the FNIH adjusts the appendicular lean mass evaluated by dual-energy X-ray absorptiometry (DXA) according to the patient's body mass index (BMI). This method is based on the largest epidemiological study with cross-validation analysis performed to date for the classification of sarcopenia 9.

Several techniques can be used for muscle mass evaluation, such as DXA, bioimpedance analysis (BIA), magnetic resonance imaging (MRI) and computed tomography (CT) 3. Computed tomography (CT) is considered one of the gold standards, but it exposes the patient to ionizing radiation and is expensive and of limited availability 10. An alternative could be the ultrasonography (US), an imaging method that has experienced extraordinary technological advances in recent years and has the advantages of low cost, high portability and bedside use. It allows evaluation of specific muscles and has proven to be valid and reliable when evaluating muscle mass, yielding results comparable to CT 11,12.

Due to the aging population and consequent increase in the prevalence of sarcopenia, it is essential to assess new methods to evaluate muscle mass, ideally methods that have a lower cost and that can be performed at the bedside. Therefore, the purpose of this study was to evaluate the validity and reliability of measurement of rectus femoris cross-sectional area (RF_CSA_) by US compared to CT for the diagnosis of sarcopenia in pre-dialysis CKD.

## MATERIALS AND METHODS

This was a cross-sectional, convenience sample-based study that evaluated 100 pre-dialysis CKD patients aged 65 years or older, of both genders, from the CKD outpatient clinic of the Hiperdia Minas Center in Juiz de Fora, Brazil. This was an additional analysis of the same population previously described in another study by our group 2. Data were collected between September 2014 and March 2016. The study was approved by the local ethics committee, and each patient signed an informed consent form. Patients who had difficulty in performing the necessary muscle strength and functional tests were excluded, along with those with severe neuropathy, liver disease, stroke sequelae, arthritis, arthrosis, amputations, severe chronic obstructive pulmonary disease (COPD), Parkinson's disease, cancer, or acquired immune deficiency syndrome (AIDS) 2.

Diagnosis and classification of CKD were based on the Kidney Disease Improving Global Outcomes (KDIGO) criteria 13 and the glomerular filtration rate was estimated from the serum creatinine levels using the CKD Epidemiology Collaboration (CKD-EPI) equation 14. As described previously, systemic hypertension was defined as a systolic blood pressure (BP) equal to or greater than 140 mmHg and a diastolic BP equal to or greater than 90 mmHg or the use of antihypertensive medications 2,15. Diabetes mellitus was defined as fasting blood glucose greater than or equal to 126 mg/dL or the use of antiglycemic drugs 2,15.

Blood samples were collected after 12 hours of fasting for evaluation of blood count and creatinine 2.

Sarcopenia was defined according to the FNIH Sarcopenia Project criteria 9. After 12 hours of fasting, patients underwent DXA using GE Lunar Prodigy Primo equipment to evaluate muscle mass and establish the prevalence of sarcopenia according to the FNIH definition 2,9. Low muscle mass was defined based on the Appendicular Lean Mass Index (ALMI), with the following formula: appendicular lean mass divided by BMI. Muscle mass was considered low when the ALMI was below 0.789 for males and 0.512 for females 2,9.

Handgrip strength was used to evaluate muscle strength, with a cutoff point under 26 kg for males and under 16 kg for females. Muscle performance was evaluated by calculating the usual walking speed over a distance of 3 meters, and a speed lower than 0.8 m/s indicated low performance 9.

Sarcopenia was defined as the presence of low muscle mass with reduced muscle strength or performance.

**Figure 1:**
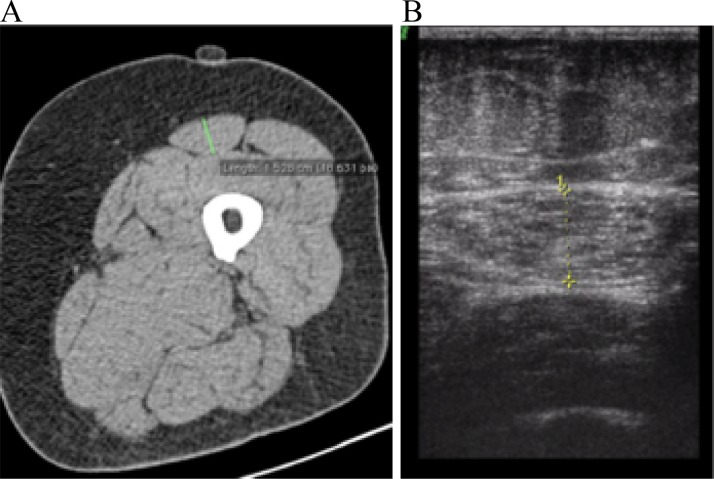
RF_CSA_ evaluation by CT (A) and US (B). RF_CSA_: Cross-sectional area of the rectus femoris; CT: Computed tomography; US: Ultrasound. Continuous green line: Rectus Femoris diameter as measured by CT; dotted yellow line: RF_CSA_ as evaluated by US

Evaluation of RF_CSA_ using US and CT was performed by a single experienced examiner. RF_CSA_ was measured by B-mode ultrasound on a Siemens Sonoline G40 (Korea, 2007) device using a 6-12 MHz linear transducer. The transducer was placed perpendicularly and transversely to the long axis of the thigh with excessive use of contact gel and minimum pressure to avoid muscle compression. RF_CSA_ was measured at the midpoint between the anterior iliac spine and the upper lateral epicondyle of the femur. The measurements were performed on the dominant leg, with the patient in supine position, with legs extended and relaxed and toes pointing to the ceiling. The dominant leg was determined to be the leg used to climb the first rung of a ladder. A set of three measurements was taken, including one at the midpoint and the others 1 cm above and below this point. RF_CSA_ was measured by placing the cursor on its inner edge, immediately below the external and internal muscle fascia in all patients (Figure 1).

Tomographic images were obtained using a Siemens Emotion device (Germany, 2007). RF_CSA_ measurements were performed on the same leg and at the same point marked during the US evaluation, with the patient lying supine. The slices were 0.63 cm thick and were acquired 1 cm above and 1 cm below the predetermined midpoint with a low-radiation protocol (Figure 1). The CT measurement data sets were analyzed in a blinded and randomized manner.

### Statistical analysis

All statistical analyses were performed using SPSS software version 21.0, with a statistical significance threshold of *p*<0.05.

In accordance with well-established methods, the results were expressed as the mean and standard deviation for continuous variables unless otherwise specified. Categorical variables were expressed as percentages.

A paired t-test was used to evaluate the difference between the RF_CSA_ means as evaluated by US and CT, and the correlations between the two methods and clinical variables were evaluated using Pearson's correlation coefficient.

Bland-Altman plot analysis 16 was used to evaluate agreement between RF measurements performed by US and CT. The Y-axis shows the difference between RF_CSA_ measurements by US and CT, and the X-axis shows the mean CT and US observations. The mean of the differences as well as the 95% confidence interval for differences are also presented.

Receiver operating characteristic (ROC) curve analysis was used to establish a cutoff point for reduced muscle mass based on the RF_CSA_ as evaluated by US. The area under the curve (AUC) was used to calculate the sensitivity, specificity and negative predictive value (NPV) of the selected cutoff points according to gender.

## RESULTS

The mean age of the studied population was 73.5±9.22 years, and 59 patients were female. Most patients were in CKD stages 3B (n=37) and 4 (n=29). All patients had hypertension and 54 had diabetes.

The clinical and laboratory characteristics of the study population are shown in Table 1.

**Figure 2:**
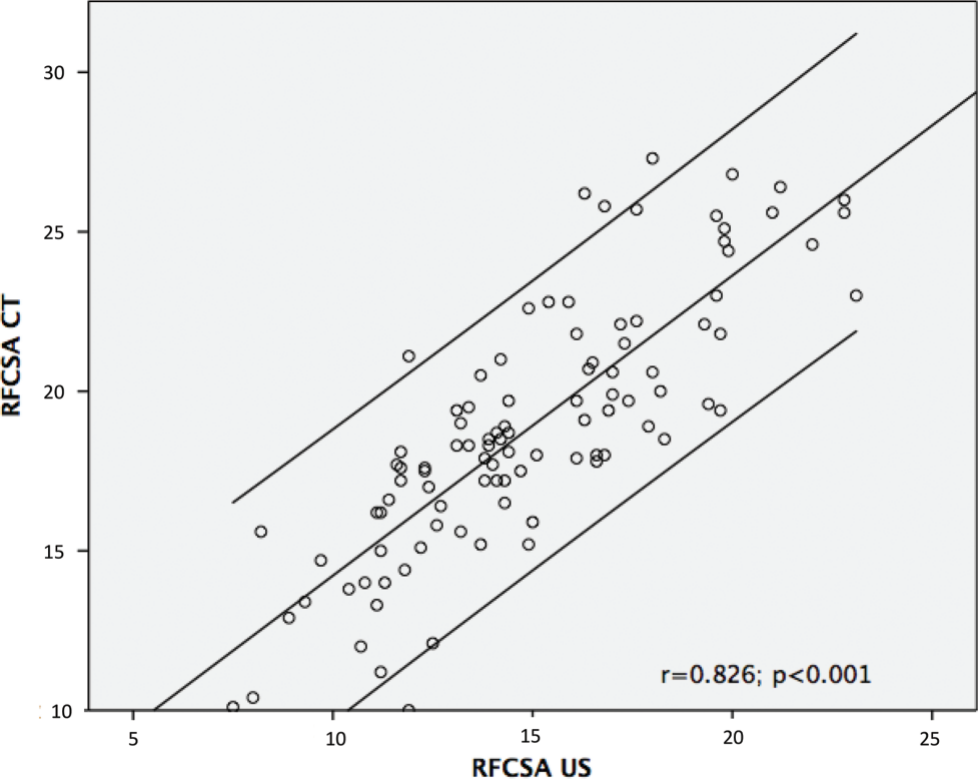
Pearson correlation coefficient of US compared to CT. RF_CSA_: Rectus femoris cross-sectional area; CT: Computed tomography; US: Ultrasound. The upper and lower lines represent the 95% confidence intervals.

**Figure 3:**
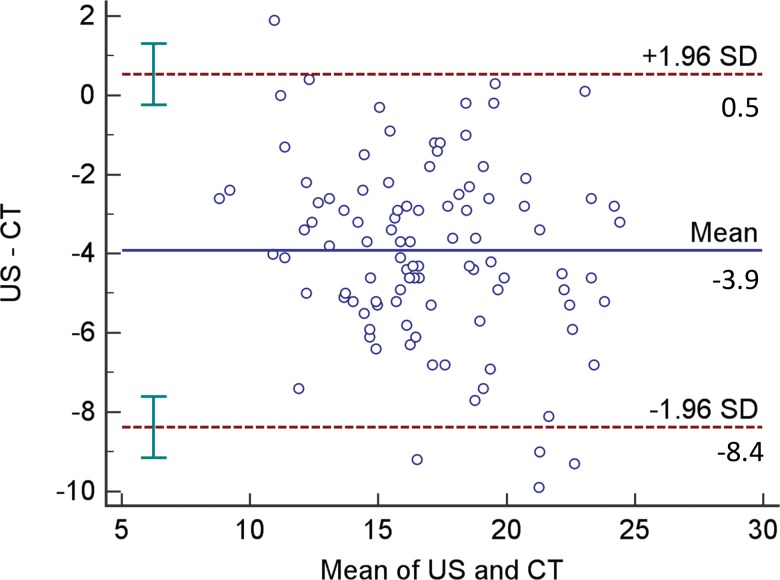
Bland-Altman plot of the differences between RF_CSA_ measurements by CT and US. RF_CSA_: Cross-sectional area of the rectus femoris; CT: Computed tomography; US: Ultrasound; SD: standard deviation. The bars represent the confidence interval limits for mean and agreement limits.

The difference in the mean RF_CSA_ as evaluated by CT and US was 3.92 mm (*p*<0.001). The Pearson correlation coefficient of US compared to CT measurements was 0.826 (*p*<0.001) (Figure 2). The Bland-Altman plot analysis showed good agreement between the CT and US measurements (Figure 3).

Our results show correlations of RF_CSA_ as evaluated by US with muscle strength (r=0.300; *p*=0.002), lean body mass in the upper limbs (LBMUL) (r=0.286; *p*=0.004), and lean body mass in the lower limbs (LBMLL) (r=0.271; *p*=0.006).

The cutoff for reduced muscle mass, based on US-measured RF_CSA_ as defined by the ROC curve was 13.2 mm, with an AUC of 0.595 for males. The cutoff for females was 10.9 mm with an AUC of 0.619. The sensitivity, specificity, and NPV of the measurements are shown in Table 2.

The prevalence of sarcopenia was 29% according to the FNIH definition, using DXA to evaluate muscle mass. Our study population had a 12% prevalence of sarcopenia based on the criterion for low RF muscle mass as assessed by US. The prevalence was 8.3% (n=1) in stages 2 and 3A of CKD and 91.6% (n=11) in stages 3B, 4 or 5 (*p*<0.001).

## DISCUSSION

Our study demonstrated that US can be used as a reliable and valid method for the evaluation of muscle mass by measuring the RF_CSA_ in pre-dialysis CKD. Our results demonstrate that sarcopenia can be diagnosed with a rapid imaging test that is simple to perform, easily interpreted and available at the bedside.

Sarcopenia is associated with high morbidity and mortality in healthy older adults and patients with chronic diseases 17 and is one of the main risk factors for the development of frailty, falls, and fractures 18. The tests considered gold standards for muscle mass evaluation and the diagnosis of sarcopenia, such as CT and DXA, are costly, of limited availability, require trained personnel, and expose the patient to radiation. Therefore, new methods need to be developed to facilitate the diagnosis of sarcopenia.

Published data show that US can be used to assess muscle mass in various situations. In 1994, Abe and colleagues suggested that US could be used for the clinical evaluation of muscle mass 19, and a few years later they published predictive equations based on US for total and regional skeletal muscle mass evaluation in healthy Japanese subjects 20. In a recent publication, Scott and colleagues found that US was a valid method for monitoring atrophy and hypertrophy of the quadriceps and atrophy of the gastrocnemius 21.

This association is also described in patients with chronic diseases. Two studies have shown that US is a valid and reliable method for evaluating muscle mass in patients with COPD 22 and coronary artery disease 23.

Regarding CKD, two studies have used CT for quantification of muscle mass and function in patients with end-stage renal disease (ESRD) and on hemodialysis 24,25. Another study also evaluated muscle mass by CT and assessed its association with inflammatory markers in patients with ESRD 26. However, to date, there is no published data on the use of US to evaluate muscle mass in pre-dialysis CKD stages, which makes our study the first to show that this method is valid and reliable for the evaluation of muscle mass in this population of patients.

In the general population, loss of both muscle mass and muscle strength are related to unfavorable outcomes 27. In CKD patients, Chang and colleagues showed that muscle strength was an independent predictor of outcomes. Patients with better handgrip strength showed better kidney survival, and interventions aimed at affecting loss of muscle strength could contribute to improvements in PEW, which is associated with worse outcomes in this population 28. Our study demonstrated an association between RF_CSA_ as evaluated by US and muscle strength, as well as LBMUL and LBMLL, suggesting that US would also be a useful method for the evaluation of PEW in patients with CKD on conservative treatment.

Once it has been shown that muscle mass can reliably be determined by US, it is important to establish cutoff points to be used in the diagnosis of sarcopenia. An Italian study established cutoff points of 20 mm for males and 16 mm for females for low muscle mass at specific muscle sites in healthy elderly individuals using US 29. In the present study, which evaluated elderly patients in pre-dialysis CKD stages, the cutoff values of RF_CSA_ were 13.25 mm for males and 10.95 mm for females. These values, along with the cutoff points recommended by the FNIH for muscle strength and performance, allowed us to establish a prevalence of sarcopenia of 12% in the studied population.

There is a well-established relationship between the prevalence of sarcopenia (as measured with traditional methods) and worsening of kidney function, and we have demonstrated this in a previous study in which we used the FNIH sarcopenia criteria and DXA to evaluate muscle mass in pre-dialysis CKD 2. In the present study, we also observed a higher prevalence of sarcopenia among individuals with more advanced CKD stages, using US to evaluate muscle mass.

Our study has limitations. The number of patients included and the fact that the study was cross-sectional in nature limited data analysis. Another important limitation was the elderly population included, which may have influenced the prevalence of sarcopenia, since there is an age-related progressive loss of muscle mass. However, from our point of view, the main limitation was the non-inclusion of a population of young healthy subjects to establish cutoffs that could be used to define low muscle mass based on US measurement in the elderly population that was evaluated.

In conclusion, our results suggest that US-based estimation of the RF_CSA_ can be used by health professionals as a valid and reliable method to evaluate muscle mass in patients with CKD on conservative treatment, as it is associated with muscle strength and mass. This finding overcomes a major problem in the diagnosis of sarcopenia, which is the definition of muscle mass; until recently assessment of muscle mass required costly methods to which most patients have limited access. However, further studies involving a larger number of participants and a control group of healthy young individuals are needed for a more definitive analysis of the use of this imaging modality for the evaluation of muscle mass in CKD patients.

The study was approved by the Ethics and Research Committee of the Federal University of Juiz de Fora and signed informed consent was obtained from all study participants prior to data collection.

## AUTHOR CONTRIBUTIONS

Souza VA was responsible for the investigation, methodology, manuscript writing, data curation and formal analysis. Oliveira D was responsible for investigation, methodology and data curation. Cupolilo EN, Miranda CS, Mansur HN and Bastos MG were responsible for the investigation and methodology. Colugnati FA was responsible for the data curation and formal analysis. Fernandes NM was responsible for the investigation, methodology, data curation and formal analysis.

## Figures and Tables

**Table 1:** Clinical and laboratory characteristics of the study population.

Age (years)	73.5±9.22
Sex	
Female	59
CKD stages	
2 and 3A	26
3B	37
4	29
5	8
Systemic Hypertension	100
Diabetes mellitus	54
Systolic blood pressure (mmHg)	152.0±25.74
Diastolic blood pressure (mmHg)	87.0±13.49
Total lean mass (kg)	41.8±8.67
ALM (kg)	17.8±3.89
ALMI (kg/BMI)	0.6±0.16
ALM in the upper limbs (kg)	4.6±1.23
ALM in the lower limbs (kg)	13.2±2.75
Handgrip (kg)	27.0±9.42
Walking speed (m/s)	0.8±0.48
Hemoglobin (g%)	12.4±1.75
Creatinine (mg/dL)	5.2 (1.00-6.24)
eGFR (ml/min/1.73 m^2^)	35.9±16.01

CDK: chronic kidney disease; ALM: appendicular lean mass; ALMI: appendicular lean mass index; eGFR: estimated glomerular filtration rate. Data are expressed as the mean±standard deviation, median (minimum-maximum), or n.

**Table 2:** Cutoff of the US-measured RF_CSA_, sensitivity, specificity, and NPV by gender.

	RF_CSA_	Sensitivity	Specificity	NPV
Males	13.2 mm	90%	70%	67.74% (95% CI 48.63 to 83.32)
Females	10.9 mm	94%	85%	67.31% (95% CI 52.89 to 79.67)

RF_CSA_: Rectus femoris cross-sectional area; NPV: Negative predictive value; US: Ultrasound; CI: Confidence interval.
